# Establishment and Characterization of Two Novel Cholangiocarcinoma Cell Lines

**DOI:** 10.1245/s10434-019-07649-5

**Published:** 2019-07-29

**Authors:** Yanhua Zhang, Jingfeng Luo, Xue Dong, Fang Yang, Miaofeng Zhang, Juanjuan Zhao, Qiangfeng Wang, Fei Zhou, Jihong Sun, Xiaoming Yang

**Affiliations:** 1 Department of Pathology, Sir Run Run Shaw Hospital, Zhejiang University School of Medicine, Hangzhou, Zhejiang China; 2 Department of Radiology, Sir Run Run Shaw Hospital, Zhejiang University School of Medicine, Hangzhou, Zhejiang China; 3 Department of Orthopaedics, Second Affiliated Hospital, School of Medicine, Zhejiang University, Zhejiang, Hangzhou China; 4 Department of Cardiology, Biomedical Research (Therapy) Center, Sir Run Run Shaw Hospital, School of Medicine, Zhejiang University, Hangzhou, Zhejiang Province, China; 5 Department of Oncology, The First Affiliated Hospital, School of Medicine, Zhejiang University, Hangzhou, China; 6 Image-Guided Bio-Molecular Intervention Research, Department of Radiology, University of Washington School of Medicine, Seattle, WA USA

## Abstract

**Background:**

Cholangiocarcinoma (CCA) is a rare, aggressive and highly lethal tumor. The disease is very difficult to diagnose and multi-modality treatments are ineffective. To improve our understanding of the biological characteristics of CCA, and facilitate the identification of valid treatments, an in-depth characterization of two novel Chinese patient-derived primary CCA cell lines was performed.

**Methods:**

Two CCA cell lines were developed and labelled ZJU-0826 and -1125. The two cell lines were characterized with respect to phenotypic, molecular, biomarker, functional and histological properties.

**Results:**

Two novel cell lines were cultured for 2 years, and maintained for more than 100 passages. They retained their typical biliary epithelial morphology and ultrastructure. The population doubling times of ZJU-0826, and -1125 were 63.84 h and 44.73 h, respectively. The cells exhibited near-triploid karyotypes with complex structural aberrations. ZJU-1125 cells had mutations in *TP53* exons. Short tandem repeats genotyping confirmed the human origin and difference between lines. An immunophenotype analysis showed that ZJU-0826 is positive for CD44, CD29, Pdx1, CD236, FoxA1, FoxA2, and Nanog, and ZJU-1125 positive for CD44, CD29, CD133, Pdx1, FoxA1, FoxA2, and Nanog. ZJU-1125 had greater invasion ability in vitro and tumorigenicity than those of ZJU-0826.

**Conclusions:**

Our results confirm the validity of the ZJU-0826 and -1125 as representative models for the elucidation of the molecular pathogenesis of perihilar CCA, and intrahepatic CCA in both in vitro and in vivo studies, respectively.

**Electronic supplementary material:**

The online version of this article (10.1245/s10434-019-07649-5) contains supplementary material, which is available to authorized users.

## Background

CCA is the most common epithelial cell malignancy within the biliary tree and the second most common primary liver cancer behind hepatocellular carcinoma.^1^^,^^2^ Hepato/choledocholithiasis, hepatitis B and C infection, liver cirrhosis, obesity, diabetes mellitus, congenital hepatic fibrosis, Caroli’s disease, and primary sclerosing cholangitis are associated with the development of CCA.^2^^,^^3^ Depending on the CCA anatomical location, it is classified as intrahepatic (ICC), perihilar, and distal CCA.^4^^,^^5^ Extrahepatic CCA accounts for near 75% of cases and the remaining 25% are intrahepatic CCA.^6^–^8^ Pathological inspection shows that most CCA are well, moderately, and poorly differentiated adenocarcinomas, and other histological subtypes are rarely diagnosed.^9^ Patients with unresectable disease have a poor prognosis; the survival time is less than 12 months following diagnosis.^5^

Although management currently combines multiple modalities including surgical treatment, radiation and systemic chemotherapy for all subtypes, long-term survival is poor. Highly desmoplastic and microenvironment-extensive tumours as well as profound genetic heterogeneity contribute to therapeutic resistance.

Reliable laboratory-based models are needed to characterize the biological features of CCA. In this study, we report the successful establishment and comprehensive characterization of two cell lines (ZJU-0826, and -1125). The cells have different biological properties and expression patterns in vitro and tumorigenicity in vivo, providing a basis for future investigations of the differences and the identification of therapeutic approaches for CCA.

## Materials and Methods

### Patients

**Fig. 1:**
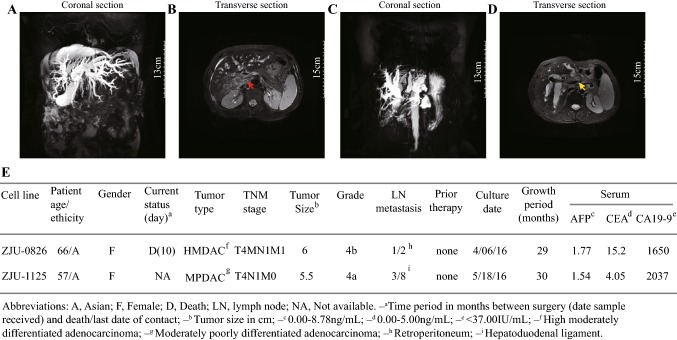
Preoperative imaging of ZJU-0826 and -1125 cells. MR imaging showed that the patient from which ZJU-0826 cells were obtained had a dilated intrahepatic bile duct that ends abruptly at the biliary confluence (**a**) and the tumour was located in the hair of the right liver (red arrow) (**b**). MR imaging demonstrated that the patient from which ZJU-1125 cells were derived exhibited a “beak sign” on the common bile duct (**c**) and the tumour occupied the perihilar bile duct (yellow arrow) (**d**). Clinical and pathological profiles of two patients with CCA used for the initiation of cell lines (**e**)

The Sir Run Run Hospital of Zhejiang University provided patient tumour samples, and written informed consent was obtained from each patient. ZJU-0826 was derived from a 65-year-old woman with perihilar CCA in 2016. The patient suffered from repeated pain with no obvious inducement, on the right side in the first half of the year. Eventually, the skin, eye, and urine changed to yellow. On admission, serum levels of CEA and CA19-9 were elevated. Magnetic resonance (MR) cholangiopancreatography demonstrated dilation of the intrahepatic bile duct and the common bile duct was unclear (Fig. 1a). A tumour was detected in the right hepatic portal (Fig. 1b, red arrow). A radical operation was performed and indicated a highly or moderately differentiated adenocarcinoma with the following TNM classification: stage IVB, T4N1M1.

ZJU-1125 was derived from a 57-year-old woman with intrahepatic CCA in 2016. The patient suffered from abdominal pain, but no other symptoms were observed. Only CA19-9 levels were outside of the reference range. MR cholangiopancreatography and pathological examinations were conducted for diagnosis. The patients did not receive any treatment prior to surgery. MR cholangiopancreatography showed a “beak sign” on the common bile duct (Fig. 1c) and revealed a tumour in the hepatic portal (Fig. 1d, yellow arrow). A surgery specimen showed a hard and thick wall. Pathology indicated a moderately or poorly differentiated adenocarcinoma classified as stage IVA, T4N1M0. Surgery was performed.

### Establishment CCA cell lines, and cell culture

The surgically resected cholangiocarcinoma tissues were obtained immediately for cell culture. Samples were aseptically washed three times in Roswell Park Memorial Institute (RPMI) 1640 medium (Invitrogen, Carlsbad CA, USA) containing penicillin (200 U/mL), streptomycin (100 µg/mL), amphotericin B (0.0085%), and ciprofloxacin (10 µg/mL), and finely minced using scalpels into 1- to 3 mm^3^ pieces removing the blood, fat, and fibroblast connective tissue for enzymatic disaggregation. The enzymatic disaggregation method for single cell suspensions followed a previously described protocol (Miltenyi Biotec GmbH, Bergisch Gladbach, Germany). Cell viability was evaluated using Trypan Blue. Cells were seeded on 60 mm Petri dishes and maintained in RPMI 1640 medium supplemented with 10% fetal bovine serum (FBS) (Gibco, Grand Island, NY, USA), and antibiotics (Invitrogen) in a humidified 5% CO_2_ incubator at 37 °C. Contaminating fibroblasts were eliminated by differential trypsinization and differential attachment.^10^–^12^ Once the cultures were 80–90% confluent, cells were harvested after treatment with 0.25% trypsin and 0.02% EDTA solution split in a ratio of 1:2 in fresh RPMI 1640 medium with additive. For continuous propagation, the cells were passaged at regular intervals, frozen periodically, and stored in liquid nitrogen. No external growth factors or stimulatory cytokines were added during the establishment of the CCA cell lines. The cell lines were named ZJU-0826 and ZJU-1125.

### Control cell lines

MCF-10A, ZR-75-1, HCT116, SK-OV-3, HeLa, HCC9810, QBC939, RBE, HUCCT1, NCCIT and AAV293 cells were obtained from the Cell Bank of Type Culture Collection of the Chinese Academy of Sciences (Shanghai, China) in 2013–2016. MCF-10A was maintained in Mammary Epithelial Cell Growth Medium (MEGM) (Lonza, Walkersville, MD, USA). ZR-75-1, HCC-9180, QBC939, RBE and NCCIT cells were maintained in RPMI1640 medium with additives (10% FBS, and antibiotics), and supplements (1.5 g/L NaHCO_3_, 2.5 g/L glucose, and 0.11 g/L sodium pyruvate). HCT116 and SK-OV-3 was maintained in McCoy’s 5A medium with additives and 2.2 g/L NaHCO_3_. HeLa cells were maintained in MEM with additives and supplements. HUCCT1 and AAV-293 cells were maintained in DMEM with additives and 1.5 g/L NaHCO_3_. Cells were cultured in a humidified 5% CO_2_ incubator at 37 °C. The medium was renewed when a colour change was observed.

### Morphological and Transmission Electron Microscopy Examinations

Cultured cells were routinely observed for cell morphology using a phase-contrast microscope.

Ultrastructural characteristics of the established cell lines were studied by transmission electron microscopy (TEM). Cultured cells were scraped into 1.2% glutaraldehyde and 2.0% glutaraldehyde fixative for 1 h at 4 °C, washed, post-fixed with 1% osmium tetroxide dehydrated in a graded series of alcohol, and embedded in resin. Ultra-thin sections were cut, stained with uranyl acetate and lead citrate, and viewed under a HT7700 scanning electron microscope (Hitachi, Tokyo, Japan).

### DNA Content and Growth Kinetics

ZJU-0826 and ZJU-1125 cells at passages 45–49 were used. For the DNA content assay, cells in the exponential growth phase were harvested and evaluated as previously described.^13^ Normal human lymphocytes were used as an internal control. For the growth kinetics assay, 1 × 10^4^ cells/well in 5 duplicates were added to 96-well plates with 100 μL of medium, and only medium in well as a control. The growth rate was assayed for 72 h at 12-h intervals. Cell were incubated with Cell Counting Kit-8 (CCK-8; Dojindo Laboratories, Kumamoto, Japan) for 2.5 h and ultraviolet absorbance was measured at a wavelength of 450 nm. The population doubling time (PDT) was calculated using the algorithm implemented in Doubling Time software (http://www.doubling-time.com).

### STR DNA Fingerprinting

Approximately 1 × 10^4^ cells were used for Short tandem repeats (STR) profiling of 17 STR loci plus the gender-determining locus, amelogenin. (the American Type Culture Collection (ATCC), Manassas VA, USA). An ABI Prism 3500 × 1 Genetic Analyzer was used to process the samples and GeneMapper IDX v.1.2 (Applied Biosystems, Foster City, CA, USA) was used for data analysis. Appropriate positive and negative controls were used as internal standards, and the results were searched against ATCC and Deutsche Sammlung von Mikroorganismenund Zelkulturen (DSMZ) databases for each sample (ATCC sales order no. SO0146233).

### Conventional Cytogenetic Analysis

Cytogenetic analyses were performed at passage 45. The established tumour cells at the exponential phase were arrested in metaphase by treatment with 0.1 μg/mL Colcemid at 37 °C for 1 h and harvested according to standard methods. Hypotonic treatment was performed with a 0.075 M KCl solution for 20 min at room temperature. Slides of fixed cells were subjected to trypsin-Giemsa-banding to identify individual metaphase chromosomes. Images of representative chromosome sets were obtained for the karyotype analysis, and the interpretation of karyotypes was based on International System for (Human) Cytogenetic Nomenclature (ISCN) (1995). The modal chromosome number was based on an examination of 100 cells.

### TP53 Mutation Screening

To detect coding region mutations in *TP53*, ZJU-0826 and ZJU-1125 cells at passages 65 and 49, respectively, were used. Genomic DNA (gDNA) was extracted using the DNeasy Blood and Tissue Kit (Qiagen, Valencia, CA, USA), and gene fragments were amplified using a Polymerase Chain Reaction kit (PCR) with specific primers (Table 1) and Taq Polymerase. PCR products were purified and sequenced by TSINGKE Biological Technology (Zhejiang, China). The sequencing traces of amplified fragments were aligned to references using Chromas (1.1.0.1) and analysed.

**Table 1:** Primer sequences for the amplification of TP53 exons^14^

Target	Forward primer (5′ → 3′)	Reverse primer (5′ → 3′)	Product (bp)	Codon position
Exon-1	GTCGGCGAGAATCCTGACT	CCAACAATGCAACTCCTATGAT	481	0
Exon 2	TCAGACACTGGCATGGTGTT	GGGGACAGCATCAAATCATC	498	1–25
Exon 3	TCAGACACTGGCATGGTGTT	GGGGACAGCATCAAATCATC	498	25–32
Exon 4	GGGACTGACTTTCTGCTCTTGT	GCCAAAGGGTGAAGAGGAATC	543	33–125
Exon 5	GTTTCTTTGCTGCCGTCTTC	TTCCTTCCACTCGGATAAGATG	387	126–187
Exon 6	TACAAGCAGTCACAGCACATGA	GGTCAAATAAGCAGCAGGTTAAGA	370	187–224
Exon 7	GTGAAACCCCGTCTCTACTGAA	GAGGAGAAGCCACAGGTTAAGA	588	225–261
Exon 8	GGAGTAGATGGAGCCTGGTTTT	GTTGGGCAGTGCTAGGAAAG	328	261–307
Exon 9	GGTAAGCAAGCAGGACAAGAAG	TACAACCAGGAGCCATTGTCTT	313	307–331
Exon 10	CATGTTGCTTTTGTACCGTCAT	TGGATACACTGAGGCAAGAATG	408	332–367
Exon 11	AACATATTTGCATGGGGTGTG	CCAGTCTCCAGCCTTTGTTC	1580	367–394

### Western Blot Analysis and Flow Cytometry

CAF, MCF-10A, ZR-75-1, HCT116, QBC939, NCCIT, RBE, HUCCT1, ZJU-0826, and ZJU-1125 cells were washed thrice with ice-cold phosphate buffered saline (PBS), lysed in RIPA buffer containing 50 mM Tris (pH 7.4), 150 mM NaCl, 0.5% sodium deoxyholate, 1% Nonide P-40, and 0.1% SDS with Pierce Protease Inhibitor Tablets (AEBSF, aprotinin, bestatin, E-64, leupeptin, and pepstain A) (Thermo Fisher Scientific Inc., Waltham, MA, USA) and separated by centrifugation. The protein concentration was detected using the BCA Protein Assay Kit (Thermo Fisher Scientific Inc). Antibodies against the following proteins were used: E-cadherin, N-cadherin, β-catenin, α-SMA, MUC1, CD146, SOX17, Vitamin D3 Receptor (VDR), pdx1, CD326, FoxA1/HNF3α, FoxA2/HNF3β, Nanog, GAPDH, and β-actin (all 1:1000, from Cell Signaling Technology, Danvers, MA, USA).

Flow cytometry was performed using a FACScan instrument (BD Bioscience, San Jose, CA, USA) and commercially available reagents for cells at passages 3–5 and 60–65. A panel of monoclonal antibodies was evaluated, including CD24, CD44, CD29, CD34, CD90, CD117, CD133, CD184, CD326, and CD338 (Biolegend, San Diego, CA, USA). Antigen expression was determined based on a significant shift in staining compared to an isotype control.

### Radiation Sensitivity Assay, and Mycoplasma Detection

For the radiation sensitivity assay, ZJU-0826 and ZJU-1125 cells at passage 83 were seeded in 96-well plates at a density of 1 × 10^4^ and subjected to radiation at 0, 2, 5, 10, or 15 Gy for 72 h. Cell viability was evaluated by measuring the absorbance of cells incubated with CCK-8 reagent for 2.5 h. The inhibition rate (%) was calculated as^12^ 100 (mean absorbance of test well/mean absorbance of control well) × 100.

The absence of mycoplasma contamination was confirmed independently using the LookOut^®^ Mycoplasma PCR Detection Kit and JumpStart™ Taq DNA Polymerase according to the manufacturer’s instructions (Sigma-Aldrich, St Louis, MO, USA). A total of 1–5 fg of mycoplasma DNA was subjected to 3% agarose gel electrophoresis for 30 min at 120 V.

### Animal Care and Subcutaneous Tumour Models

All animal experiments were approved by the Institutional Animal Care and Use Committee. For in vivo studies, ZJU-0826 and ZJU-1125 cells (1 × 10^6^) at passage 100 were subcutaneously injected into each flank of 4- to 6-week old female athymic nude mice (BALB/c nu) (SLAC Laboratory Animals Company, Shanghai, China) (*n* = 5 per group). Animals were fed in laminar flow cabinets under specific pathogen-free conditions. Tumour diameters were measured using a Vernier caliper every 5 days. After 30 days, tumours were removed and fixed in 10% phosphate-buffered formalin overnight and routinely processed for histopathology and immunocytochemistry.

### Wound-Healing Assays

ZJU-0826 and ZJU-1125 cells at passage 110 were used for the Cell Comb™ Scratch Assay according to the manufacturer’s instructions (Merck Bioscience, Darmstadt, Germany). Briefly, RIPM 1640 was replaced when the cells reached semi-confluence, and a wound was created in the cell monolayer using Cell Combs. Cells were cultured for an additional 48 h at 37 °C and 5% CO_2_. Eight scratched fields were randomly chosen cell counting.

### Migration and Invasion Assays

In vitro migration and invasiveness were evaluated as previously described.^11^ Briefly, ZJU-0826, and -1125 cells at passage 100 were seeded on a Transwell chamber (Migration) or Matrigel-coated Transwell chamber (Invasiveness) (BD Biosciences) in RIPM 1640, and migrated or invaded cells in each Transwell filter were counted in 8 randomly selected fields after 24 h and 48 h, respectively.

### Soft Agar Colony-Formation Assay

For the soft agar colony-formation assay, 10,000 ZJU-0826 or -1125 cells were cultured and evaluated as previously described.^15^

### Anoikis Assay

To determinate anoikis resistance, an anoikis assay was conducted as previously described.^16^–^18^ Briefly, 96-well plates were coated with 36.48 μg of polyHEMA in 95% ethanol (120 mg/mL) and left to dry at room temperature. Before use, coated plates were washed twice in PBS and once in Hank’s buffer. Then, suspensions of 1 × 10^5^ ZJU-0826, and -1125 cells were added to each well of the Anchorage Resistant Plate or the control 96-well cell culture plate. The cells were incubated for 72 h at 37 °C and 5% CO_2_, followed by MTT colorimetric detection.

### Histology and Immunostaining

Cells from primary and xenografted tissues were used for immunohistochemistry. For cell preparation and staining, ZJU-0826 and -1125 cells were grown for 24 h on glass coverslips, fixed with 4% paraformaldehyde and blocked with 10% normal goat serum (0.3% Triton X-100). Antibodies against the following proteins were used: CK7, CK19, vimentin, and Pan-keratin (CK-pan) for immunofluorescence, and AFP, Hepatocyte, Glypican-3, Glutamine Synthetase (GS), CK20, CAD17, CDX2, β-catenin, STAB 2, MUC1, CD146, SOX17, and Vitamin D3 Receptor for immunohistochemistry. For tissue sectioning, slide preparation, and staining, tissues were fixed in 10% formalin, embedded in paraffin, and then cut into 4-μm sections. The slides were developed with antibodies against CK7, CK19, vimentin, and Pan-keratin, and immunostained sections were counterstained with hematoxylin and assessed by microscopy.

### Statistical Analysis

Results are expressed as mean ± standard error of at least three independent experiments. Differences were analyzed with Student’s *t* tests using GraphPad Prism 7.0 (GraphPad software, Inc, La Jolla, CA, USA). Differences were considered statistically significant when *P* < 0.05.

## Results

### Primary Histopathology, and Cell Culture

**Fig. 2:**
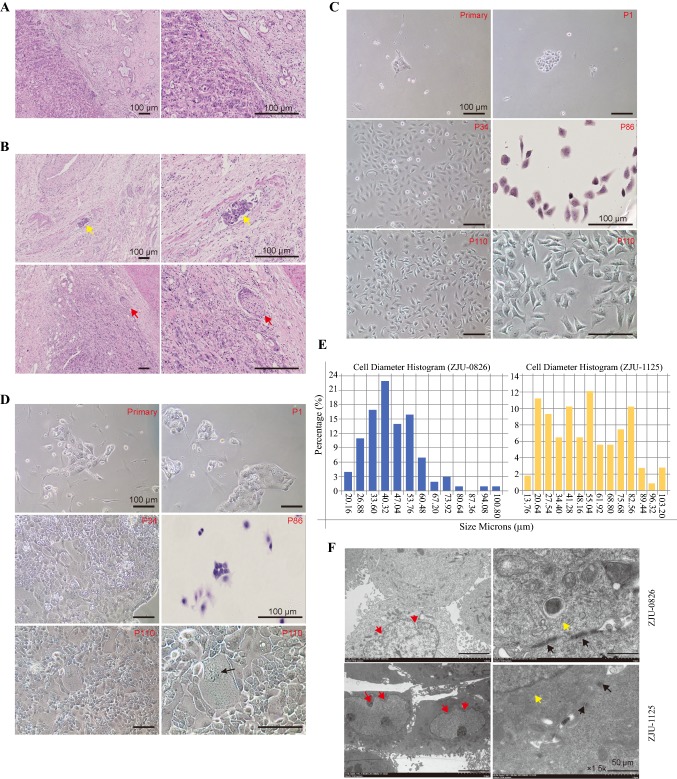
Histopathologic examination of patients and morphological features of ZJU-0826 and -1125 cells. **a**, **b** H&E staining analysis of the primary tumour from biopsy shows typical features of CCA, with medium-sized polygonal cells and poorly differentiated adenocarcinoma; in particular, the ZJU-1125 patient suffered from cancer-related embolism and nerve recidivism (**b**) (original magnification × 40 (left) and × 200 (right). Images of each cell line at different passages. In the early passages (primary and P1), all cells grew with co-existing fibroblasts. From early to late passages, the morphologic properties of cells did not change. In ZJU-0826, cells had uniform shape with a loose, polygonal-like pattern (**c**). ZJU-1125 had mixed shapes with a tight, polygonal-like pattern and occasional multinucleated giant cells (indicate by arrows) (**d**). **e** Distribution of the size (longest diameter) of ZJU-0826 and -1125 cells at passage 120. **f** Electron microscopy images. Ultrastructural features (indicated by arrows) of ZJU-0826 and -1125 cells all demonstrated indented nuclear membranes (red arrows), tonofilaments in the cytoplasm (yellow arrows) and the intercellular connections (black arrows)

Two cell lines were successfully established from two patients with advanced CCA in situ. Clinical features and pathological characteristics of the patients are shown in Fig. 1e. The patient form which the ZJU-0826 line was obtained died 10 days after surgery, and the ZJU-1125 patient did not die during the study period. A pathological analysis of the original tumor was conducted. H&E staining revealed that ZJU-0826 was a highly or moderately differentiated adenocarcinoma and ZJU-1125 was derived from moderately or poorly differentiated adenocarcinoma, the ZJU-1125 patient suffered from cancer-related embolism and nerve recidivism (Fig. 2a, b). Under a phase contrast microscope, a monolayer epithelial-pattern of substrate-adherent cells was observed, and cells showed differences in morphology and other characteristics. ZJU-0826 was morphologically homogeneous (Fig. 2c) with characteristic loose pleomorphic cells and rare multinucleated cells. ZJU-1125 was composed of polygonal and relatively uniform cells with occasional multinucleated cells, including ovoid or cuboidal cells forming a compact monolayer, and spindle-shaped cell in a loose formation (Fig. 2d). The ZJU-1125 cell line was sub-cloned by single cell sorting and seven colonies were obtained (Figure S1A–G). ZJU-0826 cells were approximately 26 to 60 μm in largest diameter, and ZJU-1125 cell diameters ranged from 13.76 to 103.20 μm (Fig. 2e). Electron microscopy showed that ZJU-0826 and ZJU-1125 cells contained many mitochondria, rough endoplasmic reticula, ribosomes, large irregular nuclei with a nuclear membrane showing deep indentation, and microvillus-like projections on the surface (Fig. 2f). CK7 and CK19 are gastrointestinal^8^ tract markers.^19^–^22^ CK-pan^23^ and vimentin^24^ are common epithelium markers. Cells and tissue were positive for CK7, CK19, and CK-pan and cells were negative for vimentin expression (Figure S2). No expression of AFP, hepatocyte, Glypican-3, GS, CK20, CAD17, CDX2, β-catenin, and SATB2 was detected (Figure S3A, B). ZJU-0826, and -1125 cell lines had higher viability and no changes in features when cultured from the cryopreserved state and were stable during a long-term culture beyond 100 passages.

### Cell Cycle, Doubling Time, and STR DNA Profile

**Fig. 3:**
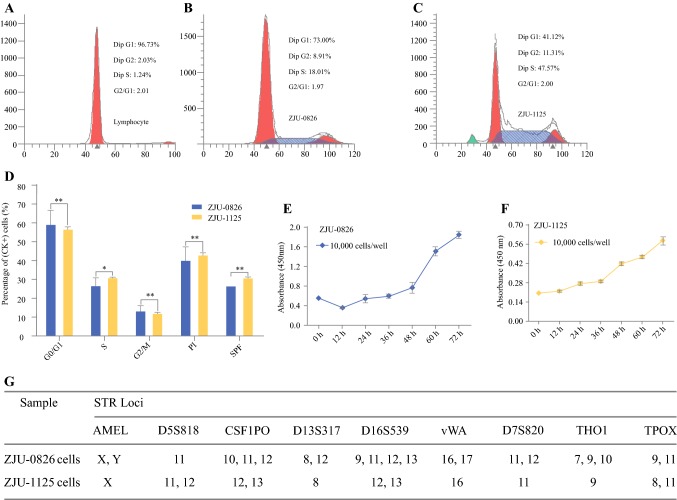
DNA content, doubling time, and immunophenotypes. DNA content and cell cycle distribution of normal lymphocytes (**a**), ZJU-0826 (**b**), and ZJU-1125 (**c**) cells and summary of a statistical analysis (**d**). Compared to ZJU-0826, ZJU-1125 had a smaller fraction in G0/G1 phase (^**^P < 0.05), an increase in the S-phase fraction (^*^P < 0.05), and a similar G2/M fraction (^**^P < 0.05), resulting in higher PI (^**^P < 0.05) and SPF levels (^**^P < 0.05). **e**, **f** Growth rate curves determined by CCK-8 showed that the PDT of ZJU-0826 and -1125 were approximately 63.84 h and 44.73 h, respectively. **g** STR DNA fingerprinting of ZJU-0826, and -1125 cells

Cells in the G0/G1 phase were diploid, in the G2/M phase were tetraploid, and in S phase were diploid. DNA content was were evaluated by propidium iodide staining and flow cytometry. Both ZJU-0826 and -1125 cells were polyploid, unlike diploid lymphocytes. ZJU-1125 cells had higher PI (*P* < 0.05) and SPF (*P* < 0.05) levels than those of ZJU-0826 cells (Fig. 3a–d). The PDTs of ZJU-0826, and -1125 were approximately 63.84 h and 44.73 h, respectively (Fig. 3e, f).

Nine STR loci were amplified by PCR-based Powerplex DNA profiling, as shown in Fig. 3g. Highly polymorphic STR DNA loci uniquely discriminated between the unrelated human cell lines and corresponding cells in the ATCC and DSMZ databases.

### Cytogenetic Analysis and TP53 Mutation Screening

**Fig. 4:**
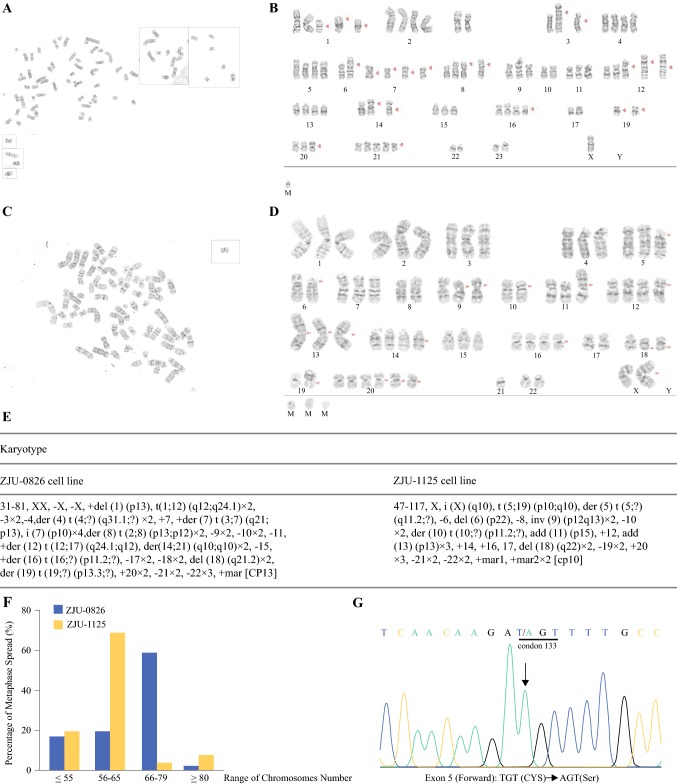
Cytogenetic analysis and *TP53* mutation screening. Representative G-banding results for ZJU-0826 (**a**, **b**) and -1125 (**c**, **d**). Representative cytogenetic abnormalities in the CCA cell lines ZJU-0826, and -1125 (**e**). Statistical analysis of chromosome number (**f**). TP53 missense mutation of in exon 5 sequenced from both ends of ZJU-1125 cells (**g**)

Conventional cytogenetic analyses of ZJU-0826, and -1125 were conducted. ZJU-0826 with near-triploid clones was characterized by a mode of 77 chromosomes; we detected deletions of chromosomes 1 and 18, increases in chromosomes 1, 7, 12, 16, and 20, two derivative chromosome 7 with for an unbalanced translocation with the long arm of chromosome 3, losses of chromosomes 3, 9,10, 11, 15, 17, 18, 21, 22 and X, a translocation only occurring on chromosome 1, and many derivative chromosomes including 4, 7, 8, 12, 14,16, and 19. ZJU-1125 with near-triploid clones was characterized by a mode of 64 chromosomes, with deletions of chromosomes 6 and 18, increases in chromosomes 12, 14, 16, and 20, losses of chromosomes 6, 8, 10, 19, 21, and 22, and derivative chromosomes 5 and 10. Representative karyotypes of ZJU-0826 and -1125 are shown in Fig. 4a–f.

A sequencing analysis of *TP53* mutations was performed using ZJU-0826 and -1125. Only ZJU-1125 showed evidence for a T > A transition (arrow) at codon 133 in exon 5, which leads to an amino acid change from Cys to Ser (Fig. 4g).

### Immunophenotypic Analysis

**Fig. 5:**
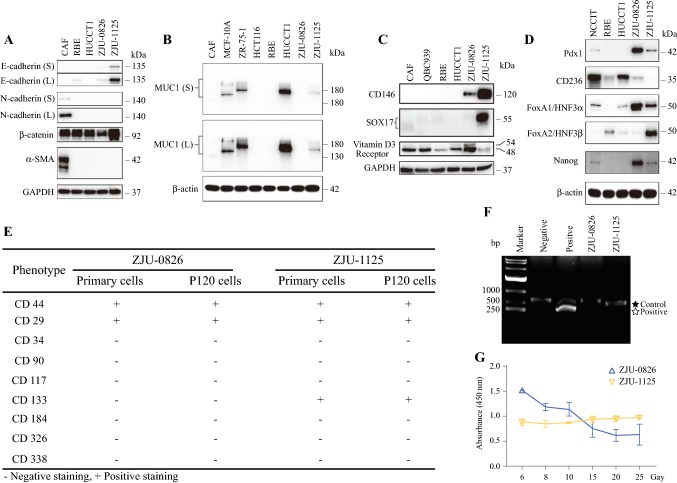
Protein expression patterns, mycoplasma contamination, and radiation sensitivity. **a** RBE, HUCCT1, ZJU-0826, and -1125 cell lines all expressed epithelial markers (E-cadherin and β-catenin), and did not express mesenchymal markers (N-cadherin, vimentin, and α-SMA). CAF cells were used as controls (E- and N-cadherin, β-catenin, vimentin, and α-SMA). **b** MCF-10A, ZR-75-1, HUCCT1, and ZJU-1125 cell lines expressed MUC1, but CAF, HCT116, RBE, and ZJU-0826 exhibited no expressed. **c** ZJU-0826 and -1125 cell lines expressed prognostic markers (poor: CD146 and SOX17. good: Vitamin D3 Receptor). CAF, QBC939, RBE, and HUCCT1 were used as controls. **d** ZJU-0826 and -1125 cell lines expressed pluripotent markers (Pdx1, Sox2, FoxA1/HNF3α, FoxA2/HNF3β and Nanog), NCCIT, RBE, and HUCCT1 cells were used as controls. **e** Immunophenotype profile of primary ZJU-0826, and ZJU-1125 cells as determined by flow cytometry. **f** Assay of mycoplasma contamination in the negative control sample, positive control sample, ZJU-0826, and -1125 cell lines by PCR. **g** ZJU-0826 was sensitive to radiation, but ZJU-1125 was not (different X-ray doses for 72 h)

Western blotting was used to examine whether the established ZJU-0826, and -1125 cells expressed epithelial, mesenchymal, and multipotential marker. RBE, HUCCT1, ZJU-0826 and ZJU-1125 all expressed epithelial markers (E-cadherin and β-catenin), but did not express mesenchymal markers (N-cadherin, vimentin, and α-SMA) (Fig. 5a, b). Compared to ZJU-1125, ZJU-0826 exhibited weaker or no expression of markers of poor prognosis (Muc1, CD146, and SOX17) (Fig. 5b, c) and expressed a marker of good prognosis (VDR) (Fig. 5c). Meanwhile the expression of markers of poor prognosis (Muc1, CD146, and SOX17) and good prognosis (VDR) in the tissue of patient’s tumor tissue were consistent with corresponding cell lines (Figure S4A, B). With respect to multipotential markers, ZJU-0826 was positive for Pdx1, CD236, FoxA1/HNF3α, FoxA2/HNF3β, and Nanog. ZJU-1125 was positive for pdx1, FoxA1/HNF3β, FoxA2/HNF3β, and Nanog and negative for CD326 (Fig. 5d).

Surface marker expression profiles were determined by flow cytometry using the original (0 < passage < 10) and late (passage 120) ZJU-0826 and -1125 cell lines. ZJU-0826 cells were positive for CD29 (small subset) and CD44 (partial) and were negative for CD34, CD90, CD117, CD133, CD184, CD326, and CD338. ZJU-1125 cells were positive for CD44, CD29, and CD133 and were negative for CD34, CD90, CD117, CD184, CD326, and CD338 (Fig. 5e).

### PCR detection of mycoplasma

Using the supernatants of ZJU-0826 and -1125 cells, a gel electrophoresis assay showed no evidence for any mycoplasma contamination (Fig. 5f).

### Radiation Sensitivity Assay

A radiation assay of the ZJU-0826 and ZJU-1125 cells was performed. Different dosages of X-ray irradiation were applied to cells for 72 h. The viability of ZJU-1125 cells did not change, unlike that of ZJU-0826 cells. The viability of ZJU-0826 cells decreased by 2/3 after exposure to high doses (20–25 Gy) (Fig. 5g).

### Tumorigenicity in Nude Mice

**Fig. 6:**
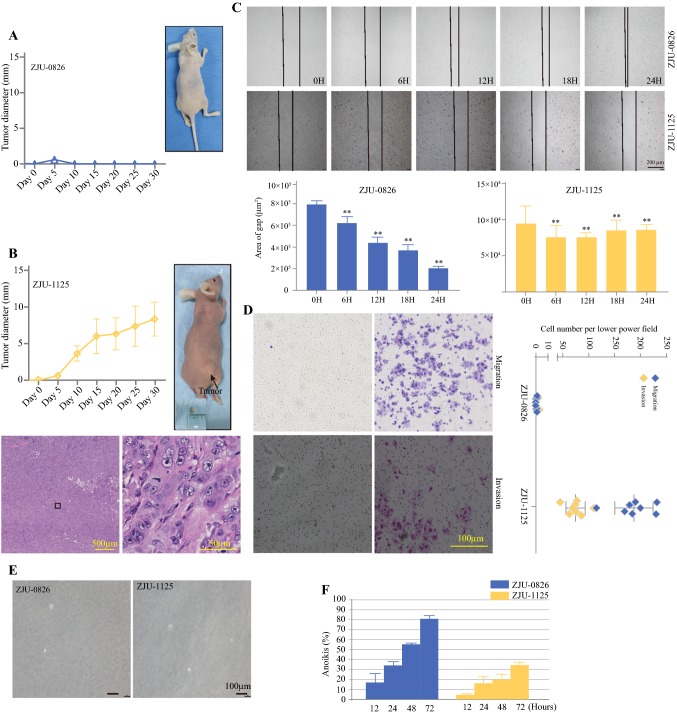
Xeno-transplantation into immunodeficient mice and Migration and Invasion property and Colony-formation assay, and Anoikis assay. **a** No tumour formation was detected in the right flanks of mice that received ZJU-0826 cells; tumour growth curve. **b** Tumour formation on the right flank of mice that received ZJU-1125 cells; tumour growth curve and H&E-stained paraffin sections of the xenograft bearing the tumour cells. **c** Representative phase-contrast images of the wound-healing assay show migrating cells in the wound margin. After 24 h, the area of ZJU-0826 cells was obviously narrow, but ZJU-1125 exhibited almost no change. **d** Representative phase-contrast images of the migration and invasion assay show a higher number of invading ZJU-1125 cells than ZJU-0826 cells. **e** After 3 weeks of culture, no colonies were observed in both ZJU-0826 and -1125 cell lines. **f** Anchorage-dependent growth was tested. Cell viability was checked by CCK-8. After 72 h, most ZJU-0826 cells lost viability, whereas the ZJU-1125 line was more resistant to anoikis

The in vivo oncogenicity of ZJU-0826 and ZJU-1125 cells was examined by intraperitoneal^25^ subcutaneous injection into BALB/c nude mice. After 30 days, all three (100%) mice treated with ZJU-0826 cells failed to developed visible tumours, while ZJU-1125 mice formed tumours that grew exponentially after the second week. In addition, there was no evidence of metastasis to other major organs. Further analysis showed that tumour masses from mice xenografted with ZJU-1125 were poorly differentiated (Fig. 6a, b).

### Behaviour of Cell Lines

The migration and invasion potential of ZJU-0826 and ZJU-1125 cells were evaluated by a healing assay, migration assay and invasion assay. In the healing assay, the gap area of ZJU-0826 deceased over time up to 72 h, but that for ZJU-1125 did not change over time. The result indicated that ZJU-0826 cells exhibit a higher migration rate than that of ZJU-1125 (Fig. 6c). Additionally, ZJU-1125 cells exhibited greater rates of migration and invasion than those of ZJU-0826 cells (Fig. 6d).

To examine the ability of tumours to grow under anchorage-independent conditions, two cell lines were evaluated by a soft agar assay. Neither cell line formed colonies after 3 weeks (Fig. 6e).

To test the resistance of ZJU-0826, and -1125 cells to anchorage-dependent programmed cell death, two cell suspensions were seeded on polyHEMA-coated plates and cultured for different durations (12, 24, 48, and 72 h). Within 72 h, most of the ZJU-0826 cells died, while ZJU-1125 cells displayed 60% viability (Fig. 6f).

## Discussion

Malignant CCA is clinically challenging owing to the sparsely cells, with borders around vital structures and high variation. These tumors account for 15% to 20% of primary hepatobiliary malignancies.^26^ The CCA incidences are highest in southeast Asia and in southern Australia. The 5-year overall survival at stages 3 and 4 are 10% and 0%, respectively.^27^

Although our understanding of CCA is growing, the precise molecular and genetic mechanisms remain largely unknown.^28^ Well-characterized cell lines have potential to be indispensable tools for determining these mechanisms. Several recent studies have reported biliary tract cancer cell lines,^25^,^29^–^39^ but comprehensive analyses of these cells are lacking. Additional validated CCA cell lines are needed to improve our understanding and develop therapies for CCA. In this study, we successfully established and characterized two novel CCA cell lines (ZJU-0826 and ZJU-1125). They were derived from a T4N1M1 cases of moderately differentiated adenocarcinoma in situ (from a 65-year-old woman with perihilar CCA) and a T4N1M0 cases of moderately or poorly differentiated adenocarcinoma in situ (from a 57-year-old woman with intrahepatic CCA). The patient from which ZJU-1125 cells were derived exhibited greater malignancy than ZJU-0826 on biopsy. STR profiles confirmed that ZJU-0826 and -1125 were different, were human-derived, and did not match any cell lines within databases (ATCC and DSMZ). Microscopic examination and immunophenotypic analyses (immunostaining and western blot) indicated typical morphological features of epithelial cells and the maintenance of stability throughout the culture period. Cell diameters microscopy, and STR analyses of ZJU-1125 suggest that it is a mixture culture of cell lines. In 1956, Johns Hopkins first reported mycoplasma contamination of HeLa cells cultured.^40^ Mycoplasma can disturb cultured cell biological and biopharmaceutical studies, and infection rates can range from 15 to 100%.^41^ In our study, ZJU-0826, and -1125 were free of mycoplasma contamination as determined by PCR. DNA content and doubling time assays revealed that ZJU-1125 cells have a faster proliferation rate than that of ZJU-0826.

Multiple structural aberrations in tumour cells are related to mispairing repair deletions (a replication error (RER)-negative phenotype),^42^ but the exact mechanisms are not known. Several studies have described CCA cells with complex numerical and structural aberrations in chromosomes.^10^^,^^21^^,^^25^^,^^28^,^43^,^44^ ZJU-0826 and -1125 cells also exhibited chromosomal abnormalities, especially the elimination of the X chromosome of ZJU-0826. The ZJU-0826 had greater chromosome loss and more derived chromosomes than ZJU-1125 cells.

Mutations in *TP53* are frequently found in human cancers and often result in amino acid substitutions.^45^ In biliary tract cancers, the role of p53 is still unclear, the mutation rate ranges from 33 to 65% depending on the anatomical site in the biliary tract.^29^ Mutations in the whole coding sequence (11 exons) were screened. In the two newly established cell lines, missense mutations were checked. Only ZJU-1125 had a mutation in codon 133 of exon 5, i.e., a TGT (Cys) to AGT (Ser) missense mutation, which might lead to a loss of DNA binding and contribute to disease pathology.

MUC1,^46^ CD146,^47^ SOX17,^48^ and VDR^49^ are common prognosis marker in CCA. We found that ZJU-1125 expresses more markers of poor and good prognosis than ZJU-0826. These result were also found in the corresponding original tumor tissues. With respect to stemness markers, only ZJU-1125 expressed CD133, a progenitor cell marker correlated with poor prognosis in CCA.^50^

The ZJU-1125 cell line had a more aggressive phenotype than that of the ZJU-0826 cell line. For example, ZJU-1125 was more tolerable to X-ray irradiation, more insensitive to detachment, exhibited greater migration and invasion in vitro, and formed solid tumours in nude mice. Meantime, the result of wound healing assay disagree with transwell, it is possible that wound healing was closely related to the result of a mixture of proliferation and migration, but transwell was just related to migration. Neither cell line formed colonies in soft agar.

## Conclusions

In conclusion, two novel CCA cell lines form Chinese women were established, named ZJU-0826, and -1125. ZJU-0826 was from one patient’s perihilar CCA, but ZJU-1125 was derived from another patient’s intrahepatic CCA. The two cell lines maintained the histological and molecular features of the primary tumor and differed from exisiting cell lines in databases (ATCC and DSMZ). ZJU-1125 cell lines had a more highly aggressive nature than ZJU-0826 in vitro (based on a doubling time assay, radiation assay, prognosis, anoikis assay, migration assay, and invasion assay) and in vivo (based on tumorigenesis in nude mice). We also first explored the effect of X-ray irradiation to CCA cell lines, and found that ZJU-0826 viability was down following the dose of X-ray, and ZJU-1125 was not affected. Accordingly, these cell lines provide a well-characterized model to investigate CCA biology and will be useful for translational and biological studies.

## Electronic supplementary material

Below is the link to the electronic supplementary material.

**Figure S1.** Morphology of ZJU-1125a–g clones and tumorigenicity. Seven clones of ZJU-1125 established by a single cell sorting technique. Phase-contrast images of cell lines showing different patterns (A–G). In a tumour formation assay using nude mice that received a transplant of cell lines, only ZJU-1125d, and exhibited no tumorigenicity (D, E). (TIFF 4689 kb)

**Figure S2.** Immunophenotypes of ZJU-0826 and -1125 cells in cytospins and tissue sections from original tumours. Cytospins and sections for the ZJU-0826 cell line were all strongly positive for bile duct epithelial markers (CK7, CK19, and CK (pan)), and did not express a mesenchymal marker (Vimentin). (TIFF 11079 kb)

**Figure S3**. Immunohistochemical analysis of ZJU-0826 and -1125 cell lines. ZJU-0826 and -1125 cells exhibited negative staining for liver markers (AFP, Hepatocyte, Glypican-3, and GS), and colon markers (CK20, CAD17, CDX2, β-catenin, and SATB2). The expression pattern confirmed that the ZJU-0826 and -1125 cell lines were all come from bile duct (A, B). (TIFF 5555 kb)

**Figure S4**. Immunohistochemical analysis of original of ZJU-0826 and ZJU-1125 tumor tissue. ZJU-0826 exhibited positive staining for CD146, and VDR, but negative for MUC1, and SOX17 (A). ZJU-1125 exhibited positive staining for MUC1, CD146, SOX17, but weakly staining for VDR (B). (TIFF 2507 kb)

## Data Availability

All data generated or analysed during this study are included in the current article. Further information is available from the corresponding author on reasonable request.
