# Supplementary figures and images for: Establishment and Characterization of Two Novel Cholangiocarcinoma Cell Lines
Source: Ann Surg Oncol. 2019 Jul 29;26(12):4134–47. doi: 10.1245/s10434-019-07649-5 (PMC6787115; doi:10.1245/s10434-019-07649-5)

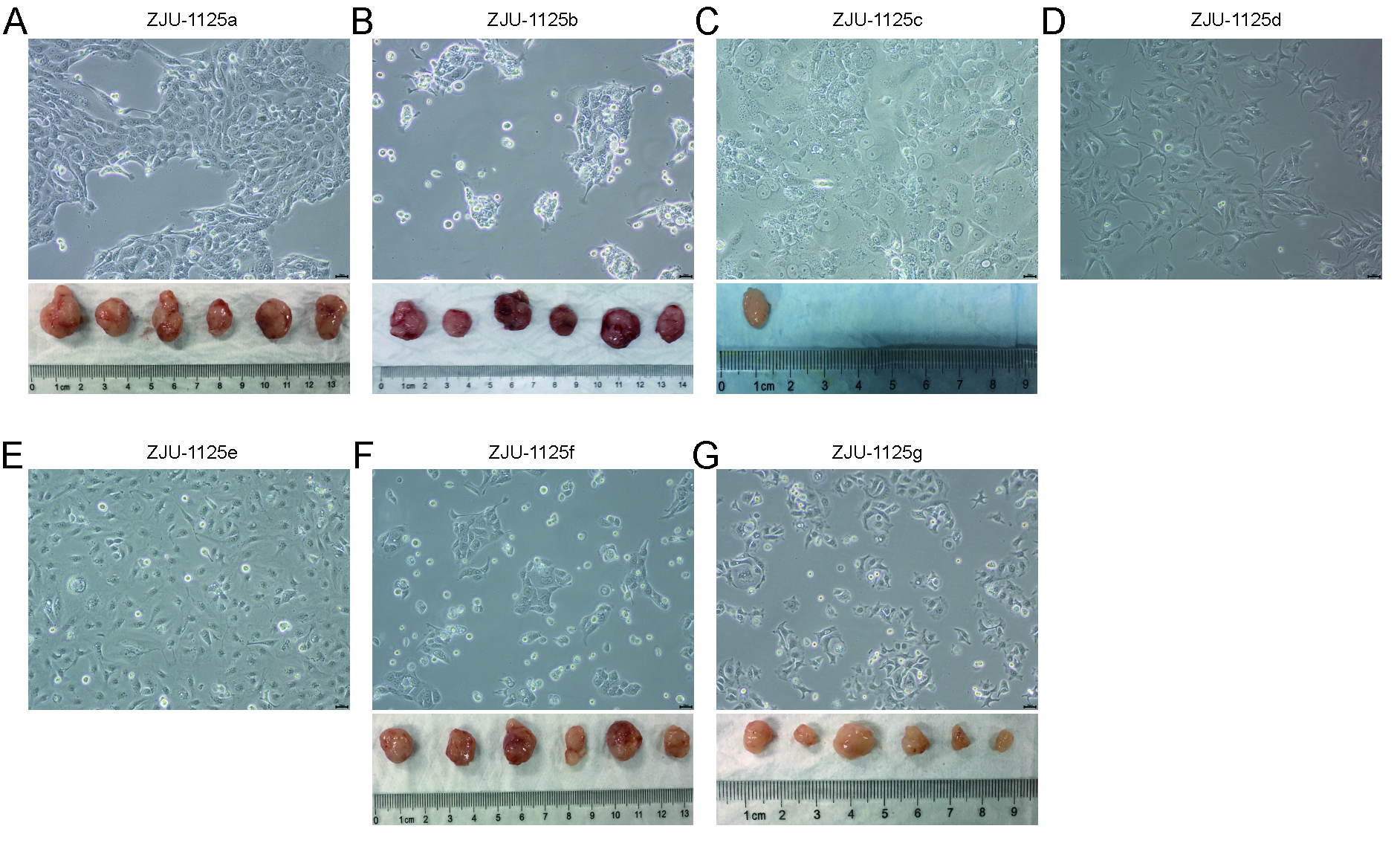

Supplement: Supplementary file 1 — Figure S1. Morphology of ZJU-1125a–g clones and tumorigenicity. Seven clones of ZJU-1125 established by a single cell sorting technique. Phase-contrast images of cell lines showing different patterns (A–G). In a tumour formation assay using nude mice that received a transplant of cell lines, only ZJU-1125d, and exhibited no tumorigenicity (D, E). (TIFF 4689 kb) [file 10434_2019_7649_MOESM1_ESM.tif]

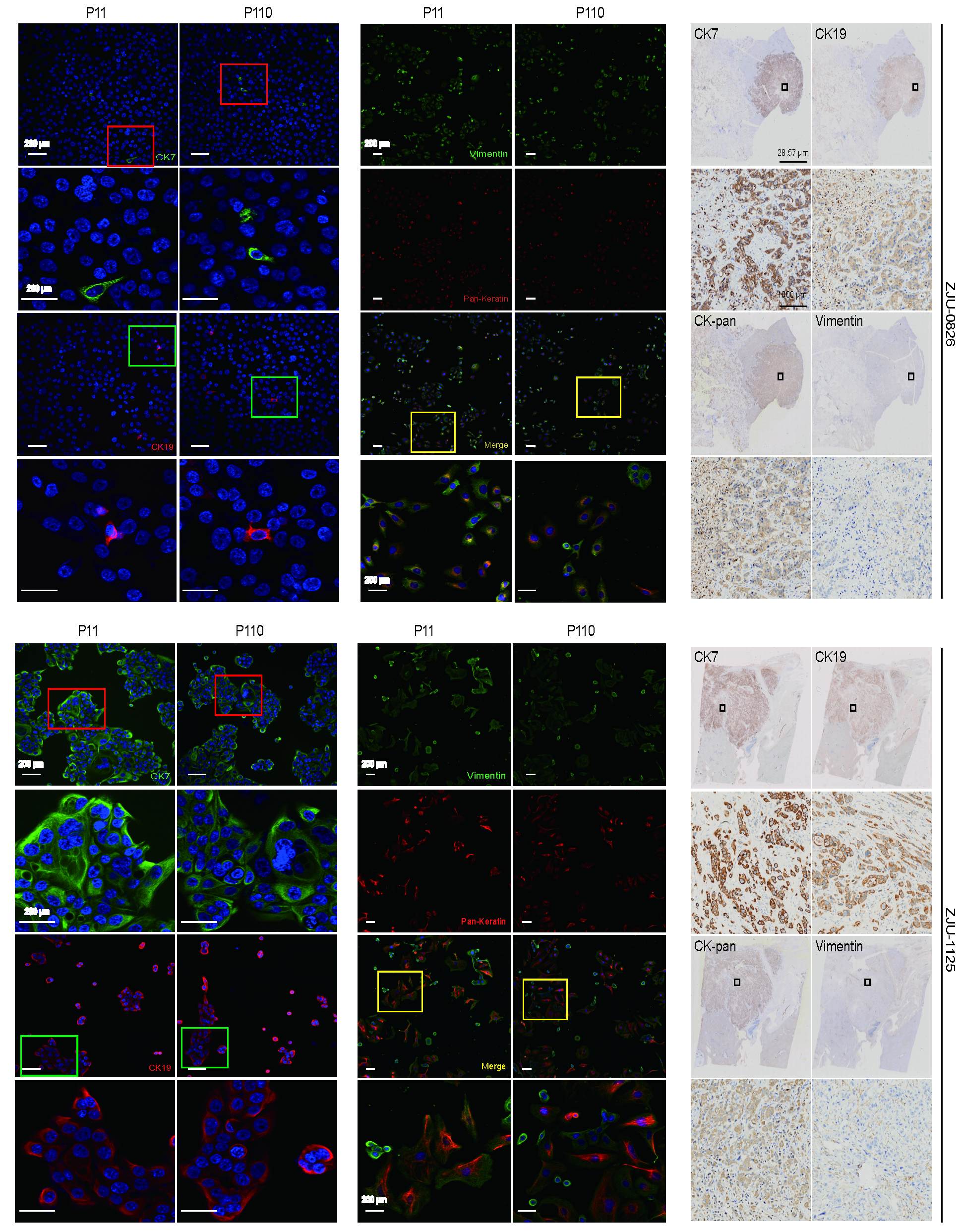

Supplement: Supplementary file 2 — Figure S2. Immunophenotypes of ZJU-0826 and -1125 cells in cytospins and tissue sections from original tumours. Cytospins and sections for the ZJU-0826 cell line were all strongly positive for bile duct epithelial markers (CK7, CK19, and CK (pan)), and did not express a mesenchymal marker (Vimentin). (TIFF 11079 kb) [file 10434_2019_7649_MOESM2_ESM.tif]

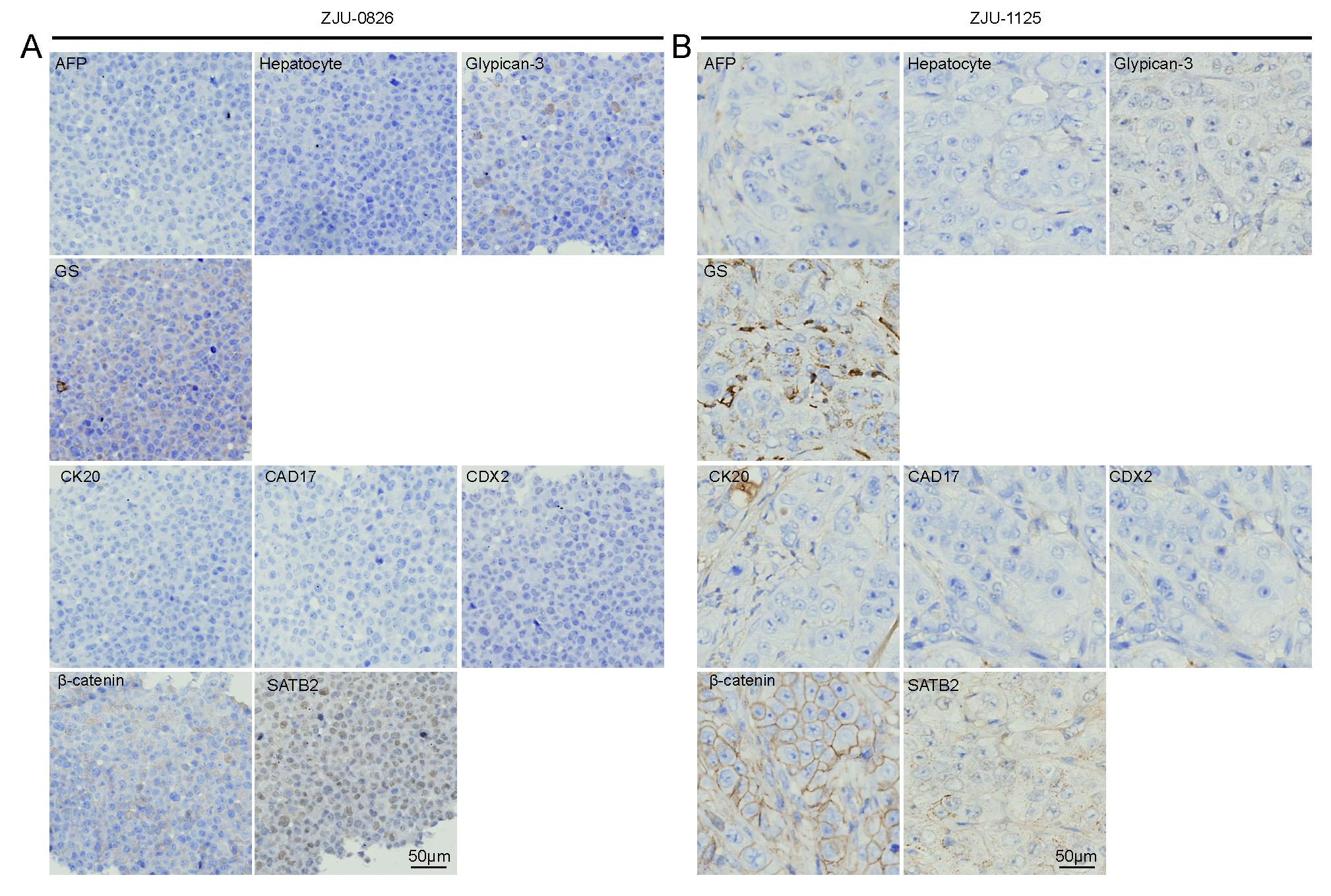

Supplement: Supplementary file 3 — Figure S3. Immunohistochemical analysis of ZJU-0826 and -1125 cell lines. ZJU-0826 and -1125 cells exhibited negative staining for liver markers (AFP, Hepatocyte, Glypican-3, and GS), and colon markers (CK20, CAD17, CDX2, β-catenin, and SATB2). The expression pattern confirmed that the ZJU-0826 and -1125 cell lines were all come from bile duct (A, B). (TIFF 5555 kb) [file 10434_2019_7649_MOESM3_ESM.tif]

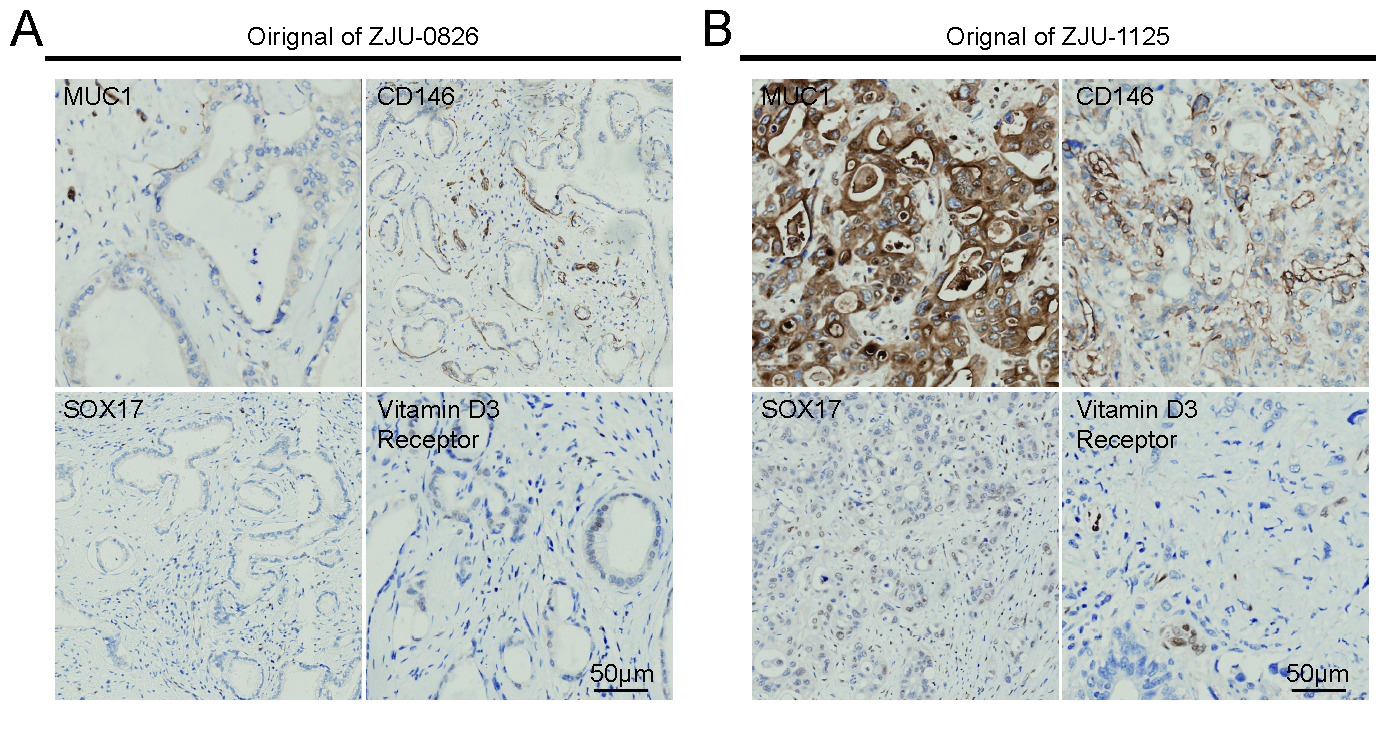

Supplement: Supplementary file 4 — Figure S4. Immunohistochemical analysis of original of ZJU-0826 and ZJU-1125 tumor tissue. ZJU-0826 exhibited positive staining for CD146, and VDR, but negative for MUC1, and SOX17 (A). ZJU-1125 exhibited positive staining for MUC1, CD146, SOX17, but weakly staining for VDR (B). (TIFF 2507 kb) [file 10434_2019_7649_MOESM4_ESM.tiff]
